# Fully Bio-Based Blends of Poly (Pentamethylene Furanoate) and Poly (Hexamethylene Furanoate) for Sustainable and Flexible Packaging

**DOI:** 10.3390/polym16162342

**Published:** 2024-08-19

**Authors:** Giulia Guidotti, Arianna Palumbo, Michelina Soccio, Massimo Gazzano, Elisabetta Salatelli, Valentina M. Siracusa, Nadia Lotti

**Affiliations:** 1 Department of Civil, Chemical, Environmental, and Materials Engineering, University of Bologna, Via Terracini 28, 40131 Bologna, Italy; giulia.guidotti9@unibo.it (G.G.); arianna.palumbo3@unibo.it (A.P.); m.soccio@unibo.it (M.S.); 2 Institute for Organic Synthesis and Photoreactivity, ISOF-CNR, Via Gobetti 101, 40129 Bologna, Italy; massimo.gazzano@isof.cnr.it; 3 Department of Industrial Chemistry “Toso Montanari”, University of Bologna, Viale Risorgimento 4, 40136 Bologna, Italy; elisabetta.salatelli@unibo.it; 4 Chemical Science Department, University of Catania, Viale A. Doria 6, 95125 Catania, Italy; vsiracus@dmfci.unict.it; 5 Interdepartmental Center for Industrial Agro-Food Research, CIRI-AGRO, Via Q. Bucci 336, 47521 Cesena, Italy

**Keywords:** bio-based polyesters, furan-based polyesters, physical blends, gas barrier properties, flexible packaging

## Abstract

In the present study, bio-based polymeric blends have been prepared for applications in the field of sustainable food packaging, starting from two furan-based homopolymers, poly(hexamethylene 2,5-furanoate) (PHF) and poly(pentamethylene 2,5-furanoate) (PPeF). PHF and PPeF were synthesized by two-step melt polycondensation—a solvent-free synthetic strategy—and then binary physical mixtures, PHF/PPeF, with different weight compositions were prepared by dissolution in a common solvent. The blends were processed into compression-moulded films, and molecular, morphological, structural, thermal, and mechanical characterizations were subsequently carried out. Blending did not negatively affect the thermal stability of the parent homopolymers, and good compatibility between them was observed. This strategy also allowed for the modulation of the chain rigidity as well as of the crystallinity, simply by acting on the relative weight amount of the homopolymers. From a mechanical point of view, the presence of PPeF led to a reduction in stiffness and an increase in the elongation at break, obtaining materials with an elastomeric behaviour. Evaluation of the gas barrier properties confirmed that the good barrier properties of PHF were preserved by blending. Finally, lab-scale composting tests confirmed a greater weight loss of the mixtures with respect to the PHF homopolymer.

## 1. Introduction

Over the years, plastic materials have become an integral part of people’s daily lives, playing a key role in the most varied applications and significantly influencing consumers’ quality of life, as confirmed by market data. Indeed, approximately 400 million tons of plastics were globally produced in 2022, 14.7% of which were destined for the European market [1,2]. This success is due to their cost-effectiveness, together with unique properties such as their easy processability, light weight, durability, mechanical strength, and thermal and chemical resistance over time [3,4]. The other side of the coin is the end of life of these materials that, if not properly disposed or recovered, accumulate uncontrollably in the environment [3]. Once deposited, plastic can break over time into smaller pollutant pieces [5,6], which eventually reach the bottom of the oceans and groundwater, thus becoming part of the universal food chain, with harmful effects on the health of living species. As reported by the *European Environment Agency*, more than 14 million tons of microplastics have been accumulated until now in the oceans [7]. All this, as well as the greater awareness of both producers and consumers on ecological issues, have led governments to take action. The European Union has started pursuing holistic policies to achieve, among the objectives, zero pollution, a reduction in greenhouse gas emissions and the transition to a circular economy by 2030, and, by 2050, the climate neutrality target [8]. Among these actions, in 2023, 17 sustainable development goals were defined as a standard for sustainable development in many fields, including healthcare, clean energy, manufacturing responsibility, and climate action [9]. Accordingly, the minimization of the volumes of plastics through reuse, recycling, and upcycling [10]; better waste management; and the use of flexible packaging instead of rigid ones, characterized by smaller volumes, are among the first actions to be taken [11,12].

These considerations are even more valid for packaging, an economic sector characterized by large volumes of plastics and relatively short life-cycles, which, alone, covers about 40% of global production [2]. As mentioned, possible solutions include recycling, which can certainly contribute to a lower consumption of virgin raw materials, but they are not always economically sustainable or practicable, especially for multilayer films or materials contaminated by food, as in the case of food packaging [13,14,15,16]. Furthermore, in developing countries where there is a lack of adequate policies and infrastructure for effective recycling, landfill accumulation is still the predominant solution [17,18,19]. For this reason, the search for alternative and bio-based sources of monomers for the eco-design of new plastics should be maximized too. The best choice fell on renewable plastic, which, through synthetic strategies with low environmental impact, can give life to 100% green materials.

Considering that bioplastics (i.e., bio-based, biodegradable, or both [20]) currently account for 0.5 percent of the global plastic produced annually [21], because of their high costs and lower performance compared to traditional petroleum-based ones [22,23], many efforts should be taken to improve their properties, making them, at the same time, economically affordable [24]. 

Within this complex framework, the aim of the present study is the realization of bio-based polymeric blends, characterized by suitable mechanical and gas barrier properties for food packaging applications. The starting materials belong to the class of aromatic polyesters, which are particularly suitable for this purpose [25,26] as they can be easily synthesized, even in the absence of solvents, and can be thermally stable, lightweight, and characterized by properties that can be modulated according to their chemical structure, to meet the requirements of different packaging materials, specifically, the homopolymers derived from 2,5-furandicarboxylic acid (FDCA), a bio-based chemical building block obtainable from 100% renewable sources, such as from the catalytic dehydration of carbohydrates [27,28]. This monomer is considered a promising eco-sustainable alter-ego of terephthalic acid, which is currently the starting building block of some of the most produced and used packaging plastics, such as PET, just to cite one. FDCA became so promising in the last few years that its market size, which was valued at 762.9 million in 2022, is expected to grow about 34% every year in the period 2023–2030 [29].

From an academic point of view, many studies have been carried out on the realization of furan-based polyesters [30,31,32,33,34], copolymers [35,36,37,38,39,40,41], and blends [42,43,44,45,46,47,48,49], thus obtaining a wide plethora of sustainable materials. Among them, blends are of particular interest, as the physical mixing technique is simple, cheap, and easily scalable on an industrial level. Moreover, the final properties are usually a combination of the original functionalities and strong points.

In the present study, three binary blends were prepared, starting from poly(pentamethylene furanoate) (PPeF) and poly(hexamethylene furanoate) (PHF), which were mixed together in different weight ratios (25, 50, and 75%, respectively). PHF is a semicrystalline and rigid material, with good gas barrier properties and not compostable [50,51]. Conversely, PPeF is rubbery and amorphous at room temperature, showing elastomeric mechanical behaviour and an impressively low permeability to gasses [50,52]. Therefore, mixing these two homopolymers together would be useful to modulate their functional properties, thus obtaining final materials with balanced mechanical properties and efficient gas barrier behaviour. These starting homopolymers have been specifically chosen for several reasons: first, by combining FDCA with two bio-based glycols, such as 1,5-pentanediol and 1,6-hexanediol, fully bio-based materials are obtained. Indeed, 1,6-hexanediol can be synthesized from hydroxymethylfurfural, which is derived from cellulose and is the same intermediate used to prepare FDCA [53], while the production of 1,5-pentanediol can be performed starting from biomass-derived tetrahydrofurfuryl alcohol, with furfural as the intermediate [54]. Also, the parent homopolymers have a very similar chemical structure, which should favour their miscibility or at least compatibility, the latter of which is fundamental in exhibiting good final functional performances of the new materials. Moreover, thanks to the similar chemical structure of PPeF and PHF, the resulting blends can be considered real monomaterials, thus making their recycling easier and more feasible. 

The blends, obtained by dissolution in a common solvent and processed into films by compression moulding, were then characterized from thermal, structural, and mechanical points of view. The phase dispersion was evaluated qualitatively by scanning electron microscopy. Lastly, the permeability to oxygen and carbon dioxide was analyzed, together with their biodegradability in compost. Thus, the present research provides new insights into the development of high-performance bio-based blends for sustainable food packaging applications. 

## 2. Materials and Methods

### 2.1. Materials

Dimethyl 2,5-furandicarboxylate (DMF) was purchased from Sarchem Labs (Farmingdale, NJ, USA), 1,5-pentanediol (PeD) from Fluorochem (Glossop, UK), 1,6-hexanediol (HD) from TCI (Zwijndrecht, Belgioum), and titanium tetrabutoxide (TBT) and titanium isopropoxide (TIP) from Sigma-Aldrich (Saint Louis, MO, USA). All the reagents were reagent-grade.

### 2.2. Poly (Hexamethylene 2,5-Furanoate) and Poly (Pentamethylene 2,5-Furanoate) Synthesis

The synthesis of poly(hexamethylene 2,5-furanoate) (PHF) and poly(pentamethylene 2,5-furanoate) (PPeF) was carried out by two-step melt polycondensation in a 250 mL glass reactor, continuously stirred (100 rpm), and put in a thermostated silicon oil bath, according to the conditions described elsewhere [51,52]. The starting monomers were DMF and HD, and DMF and PeD, respectively (glycolic molar excess of about 300%), together with 200 ppm of TBT and 200 ppm of TIP, used as catalysts. In brief, during the first step, transesterification reactions occurred under a pure nitrogen flow at a temperature of 190 °C, while methanol was distilled off (1.5 h). The second step was carried out under high vacuum (0.06 mbar) at a temperature of 200 °C, in order to eliminate the glycolic excess, favouring the increase in molecular weight. During this phase, which lasted about 2.5 additional hours, a gradual increase in the torque was observed. When this torque reached a plateau value, the synthesis was stopped and the high-molecular-weight polymers were discharged from the reactor. 

### 2.3. Molecular Characterization

The chemical structure of PHF and PPeF was confirmed by proton-nuclear magnetic resonance spectroscopy (^1^H-NMR) using an Agilent Varian Inova 400 MHz instrument (Palo Alto, CA, USA). Measurements were carried out at room temperature. Samples were prepared by dissolving about 10 mg of material in 0.7 mL of deuterated chloroform containing 0.03 vol % tetramethylsilane (TMS) as the internal standard. In the case of PHF, a few drops of trifluoroacetic acid were added to the solution just before the measurements to favour the complete dissolution of the sample. For ^13^C-NMR experiments, 40 mg of material was used instead of 10 mg; a few drops of trifluoroacetic acid were added immediately before analysis to completely dissolve the samples.

The molecular weights (M_n_) of PHF and PPeF and the corresponding polydispersity indexes (D) were evaluated at 30 °C by gel permeation chromatography (GPC) using an HPLC Lab Flow 2000 apparatus (Waters, Milford, MA, USA) equipped with a Rheodyne 7725i injector (Thermo Fisher Scientific, Waltham, MA, USA), a Phenomenex (Torrence, CA, USA) Phenogel MXM 5 μm mixed-bed column, and an RI K-2301 (KNAUER, Berlin, Germany) detector. The instrument was calibrated with polystyrene standards in the range of 550–2,500,000 g/mol. HPLC-grade chloroform was used as the eluent, with a flow of 1 mL/min. The samples were prepared by dissolving the homopolymers in the same solvent used as the eluent (2 mg/mL). In the case of PHF, a few drops of 1,1,1,3,3,3-hexafluoro-2-propanol were added to completely dissolve the sample. 

### 2.4. Blend Preparation and Processing

After synthesis and purification, binary mixtures were prepared by starting from different weight amounts of the two parent homopolymers (75/25, 50/50, and 25/75). The polymers were dissolved, under magnetic stirring at room temperature, in the minimum weight amount of the 1,1,1,3,3,3-hexafluoro-2-propanol/chloroform (5% *v*/*v*) mixture. The resulting solutions were then poured into Petri dishes to allow solvent evaporation. Samples were designated as PHF_x_/PPeF_y_, where x and y represent the relative wt% of the two parent homopolymers. 

Before being tested, samples were processed in the form of films (about 100 μm thick) by compression moulding, using a Carver (Wabash, IN, USA) C12 laboratory press. About 3 g of each blend was put in between two Teflon sheets inside the press and heated to a temperature of 190 °C. After 2 min, it was necessary to let the sample melt, and the pressure was increased to 6 ton/m^2^ and maintained for 2 further minutes. Then, the films were ballistically cooled, in press, to room temperature.

### 2.5. Morphological Characterization of the Homopolymers and of the Blends

The phase dispersion of the blends was qualitatively evaluated using a Hitachi (Tokyo, Japan) S-2400 scanning electron microscope (SEM), operating at 15 kV. The cryo-fractured cross-sections of each sample were analyzed after the deposition of a gold conductive coating.

### 2.6. Thermal and Structural Characterization of the Homopolymers and of the Blends

TGA measurements were performed on both the homopolymers and the blends by means of a Perkin Elmer (Waltham, MA, USA) TGA4000, equipped with Pyris 11 software, under a pure N_2_ flow (40 mL/min). About 5 mg of material was heated at a constant rate of 10 °C/min in the temperature range of 40–800 °C. T_onset_ was calculated as the temperature corresponding to the beginning of degradation, while T_max_ was calculated as the minimum of the thermogram derivative. 

Differential scanning calorimetry was performed using a Perkin Elmer (Waltham, MA, USA) DSC6 under pure N_2_ flow (20 mL/min). About 5 mg of each sample was placed in aluminium pans and subjected to a thermal programme controlled by Pyris 11 software: heating from −30 to 190 °C at 20 °C/min (I scan), rapid cooling from 190 to −30 °C at 100 °C/min, and subsequent heating from −30 to 190 °C at 20 °C/min (II scan). The glass transition temperature (T_g_) was calculated as the midpoint of the glass-to-rubber transition step, while the relative specific heat increment (ΔC_p_) was determined as the height between the two baselines related to the glass-to-rubber transition step. The melting temperature (T_m_) and the cold crystallization temperature (T_cc_) were calculated as the peak maximum/minimum of the endotherms/exotherms in the DSC traces, respectively, while the corresponding heat of fusion (ΔH_m_) and heat of crystallization (ΔH_cc_) were obtained from the areas of the endothermic and exothermic phenomena, respectively.

Wide-angle X-ray scattering (WAXS) experiments were carried out on films obtained from both the homopolymers and the blends using a PANalytical (Almelo, The Netherlands) X’PertPro diffractometer coupled with a copper source (λ = 0.154 nm) and equipped with a solid-state X’Celerator detector (0.1° steps, rate = 100 s/step). The crystallinity degree (X_c_) was calculated as the ratio between the crystalline diffraction area (A_c_), obtained by subtracting the amorphous halo from the total area of the diffraction profile, and the area of the whole diffraction profile (A_t_). 

### 2.7. Mechanical Characterization of the Homopolymers and of the Blends

Tensile tests were performed using an Instron (Norwood, MA, USA) 5966 dynamometer, equipped with rubber grips and a transducer-coupled 1 kN load cell. Measurements were performed on rectangular film specimens (5 mm × 50 mm), which were stretched at a rate of 10 mm/min. The gauge length was 20 mm. The instrument measures the load applied as a function of the displacement (which is calculated as the ratio between the length variation and the initial one) and can convert these data into stress–strain curves. The elastic modulus (E) was calculated as the slope of the initial linear segment of the curve without the use of any extensometer, while the stress at break (σ_B_) and elongation at break (ε_B_) were determined as the values of stress and elongation at the breakpoint. At least 6 specimens were tested for each material, and the results obtained are reported as the average value ± standard deviation.

### 2.8. Gas Permeability Measurements of the Homopolymers and of the Blends

The permeability of PHF and PPeF and the relative blends towards dry O_2_ and CO_2_ (0% relative humidity) were tested with a manometric method using a permeance testing device, type GDP-C (Brugger Feinmechanik GmbH, Munchen, Germany), in line with the Gas Permeability Testing Manual and standards ASTM 1434-82 (2009) (*Standard test method for determining gas permeability properties of plastic film and coating*), DIN 53536 (*Determination of permeability of rubber to gases*), and ISO/DIS 15105-1:2007 (*Plastic film and sheeting determination of gas transport rate; part I: differential pressure method*). Each film (area of 78.5 cm^2^) was first placed in between the two chambers of the instrument, and then the upper one was filled with the gas under investigation with a stream of 100 cm^3^/min, at room pressure. In the lower chamber, a pressure transducer measured the increase in gas pressure as a function of time. The so-obtained pressure–time data were converted into Gas Transmission Rate (GTR) values, directly related to the permeability of the polymeric film. The results obtained are reported as the mean value of at least 3 measurements.

### 2.9. Lab-Scale Composting Studies of the Homopolymers and of the Blends

Lab-scale composting tests were performed using hydrated mature compost kindly provided by “Nuova Geovis S.p.A”—HERA Group of Sant’Agata Bolognese (BO). Square pieces (1.5 × 1.5 cm) of each polymeric film were weighed, placed in between two layers of wet compost, and then put in a thermostated water bath (Julabo (Seelbach, Germany) SW22 incubator) under mechanical agitation and controlled temperature and humidity (58.0 °C and 90% RH, respectively). Periodically, sacrificial specimens were withdrawn from the compost and washed with a 70% *v*/*v* ethanol solution. Once dry, their percentage weight loss was calculated according to the following equation:

(1) $$\%\;weight\;loss=\frac{m_{i}-m_{f}}{m_{i}}\times 100$$

where *m_i_* is the initial mass of the samples and *m_f_* is their final one.

Furthermore, DSC analysis was carried out on the partially degraded films and on the blanks (i.e., films incubated for 1 month at 58 °C in a humid environment, but without compost) to verify how the permanence in compost affected the crystallinity and the main thermal transitions of the materials investigated. Lastly, the surface morphology of samples incubated for 6 months was analyzed through scanning electron microscopy (SEM).

## 3. Results and Discussion

### 3.1. Synthesis and Molecular Characterization

**Figure 1:**
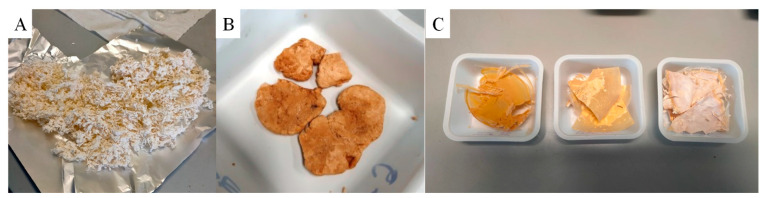
Pictures of (**A**) PHF; (**B**) PPeF; (**C**) PHF/PPeF blends.

PHF appeared in form of white flakes (Figure 1A), while PPeF was a light-coloured rubbery solid (Figure 1B). PHF/PPeF blends appeared as light-coloured solids (Figure 1C). 

^1^H-NMR analysis allowed confirmation of the chemical structure of the parent homopolymers, thus excluding the occurrence of side reactions during polycondensation. This can be assessed since the only signals in the spectra were those related to the polymers and to the solvents (Figure S1). In detail, for PHF, the three peaks of hydrogen atoms of 1,6-hexanediol ((4H, m), (4H, m), and (4H, t)) can be found at δ 1.49 ppm, δ 1.81 ppm, and δ 4.48 ppm, respectively, while for PPeF, the methylene protons of the glycolic subunit (2H, m), (4H, m), and (4H, t) were located at δ 1.55 ppm, δ 1.80 ppm, and δ 4.35 ppm, respectively. In both cases, the singlet related to the furan subunit was located at lower fields, at δ 7.21 ppm for PPeF and 7.31 ppm for PHF, respectively. Also, in the spectra obtained with the ^13^C-NMR test, no further peaks apart from those related to the carbon atoms of the polymers can be observed (Figure S2).

The optimization of the synthetic process was also confirmed by GPC measurements. For both the homopolymers, the molecular weight (M_n_) was high and comparable, ranging from 28,900 g/mol for PHF to 29,600 g/mol for PPeF, with narrow polydispersity indexes (Đ) of 2.3 and 2.4, respectively, in line with the values obtained for polycondensate polymers. 

### 3.2. Morphological Characterization

**Figure 2:**
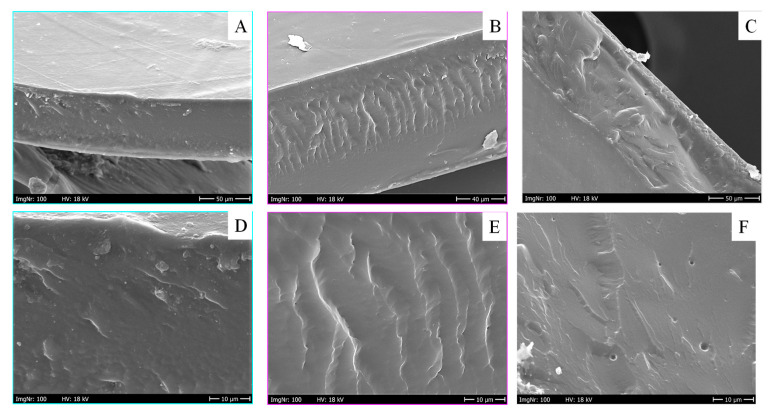
SEM micrographs of cryo-fractured cross-sections of the blends at different magnifications (upper pictures 500×, lower pictures 2000×): (**A**,**D**) PHF_75_/PPeF_25_; (**B**,**E**) PHF_50_/PPeF_50_; and (**C**,**F**) PHF_25_/PPeF_75_.

SEM micrographs of the cryo-fractured sections of the three PHF/PPeF mixtures at different magnifications are shown in Figure 2. As it can be seen, for all the blends, the two homopolymers are characterized by a very good compatibility, as most of the fracture surface is quite homogeneous and there is no clear phase separation, even at higher magnifications (Figure 2D–F). This result is particularly positive, since commonly, the presence of two phases not chemically bonded results in fracture surfaces with holes and cavities [43,48].

### 3.3. Thermal and Structural Characterization

**Figure 3:**
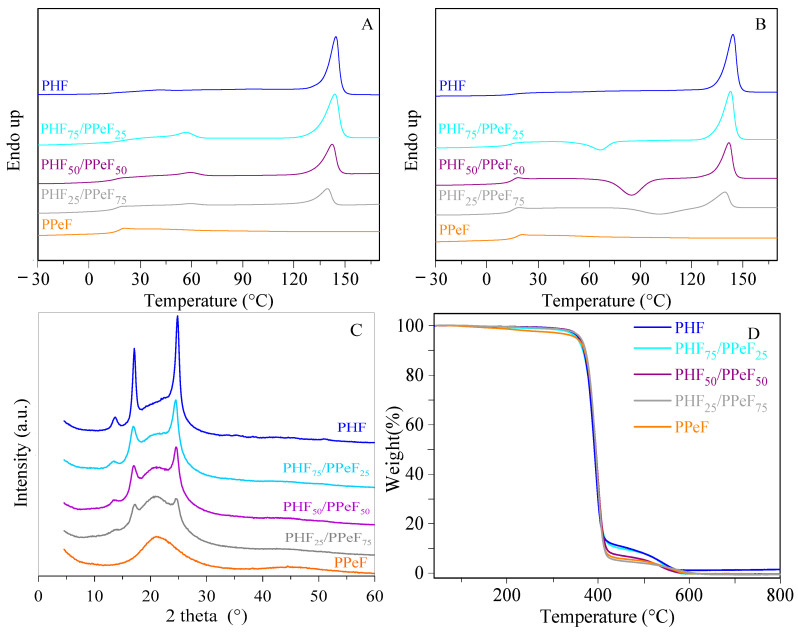
First (**A**) and second (**B**) DSC scans, WAXS profiles (**C**), and TGA curves (**D**) of PHF, PPeF, and PHF/PPeF blends.

The films obtained from the PHF/PPeF blends after three weeks from moulding were subjected to the same calorimetric treatment. The DSC profiles of the first and second scan are shown in Figure 3A and 3B, respectively, while the relative thermal data are reported in Table 1. 

In agreement with the literature data [55,56], the PHF homopolymer has a T_g_ below room temperature and a melting point at 144 °C, indicating that it is a semicrystalline polyester with a mobile amorphous phase. On the other hand, PPeF is an amorphous and rubbery polymer, with only the T_g_ jump at 16 °C being present [52,57]. As expected, all the blends exhibit an intermediate behaviour, depending on their relative composition. In detail, they are all semicrystalline, with T_m_ values slightly lower than that of the PHF, indicating a partial miscibility of the two components, in good agreement with SEM observations (Figure 2). As expected, since PPeF is not capable of crystallizing unless after very long periods of time at room temperature (6 months), the crystalline phase developed in the blend is ascribable only to PHF, and ΔH_m_ values decrease with the amount of PHF. Moreover, in all the DSC profiles, a slight endothermic phenomenon occurs in the temperature range between 57 and 59 °C, whose intensity increases with the amount of PHF (Table 1).

All the molten samples were subjected to rapid cooling and analyzed upon a second heating measurement (Figure 3B, Table 1). PPeF was completely amorphous, while the PHF profile was the same as the one observed in the I scan, indicating that, despite the high cooling rate, the polymer could not be quenched in the amorphous phase. As for the blends, the T_g_ values were all similar and close to those of the parent homopolymers. Moreover, once T_g_ was exceeded, the macromolecular chains were able to fold in an ordered structure at a higher temperature as the amount of PPeF was increased. This evidence highlights that the presence of this homopolymer, which is completely amorphous, hinders the crystallization of the PHF-rich phase. As for PHF_75_/PPeF_25_, ΔH_m_ was higher than ΔH_cc_, indicating that quenching was not effective, while for PHF_50_/PPeF_50_. and PHF_25_/PPeF_75_, the cooling rate was high enough to block the macromolecular chains in the amorphous state (ΔH_cc_ = ΔH_m_).

In order to determine their crystallinity degree (X_c_), wide-angle X-ray diffractometric analysis (WAXS) was performed. The diffraction patterns are shown in Figure 3C, while the degrees of crystallinity are reported in Table 1.

With regard to the two homopolymers, no reflections due to the crystalline phase were observed in PPeF, while the semicrystalline nature of PHF was confirmed by three intense reflections at 2Ɵ = 13.7°, 17.1°, and 24.8°. All the PHF/PPeF blends had reflections in the same position as those of PHF, confirming that the crystalline phase remained the same, regardless of the amount of PPeF present. However, their intensity, and, in turn, X_c_ values, became lower as the amount of PPeF was increased, in agreement with the calorimetric data (Table 1). 

To determine the thermal stability of the homopolymers and the blends, thermogravimetric analysis (TGA) was performed under a controlled nitrogen atmosphere. The thermograms are shown in Figure 3D, while the relative onset temperature (T_onset_) and the temperatures corresponding to the maximum degradation rate (T_max_) are listed in Table 1. 

As for PHF and PPeF, it is worth noticing that their thermal stability is particularly high and comparable, with T_onset_ being above 370 °C and T_max_ above 390 °C, in line with data obtained from previous studies [51,52,56]. Moreover, degradation occurs in two steps, with the thermograms being characterized by a shoulder when a temperature of 400 °C is reached. At the end of the measurements, weight loss is complete. As expected, the mixtures show a similar behaviour as their reference homopolymers, confirming that blending does not alter the high stability of the starting materials, which is one of their strong points. 

### 3.4. Mechanical Characterization

**Figure 4:**
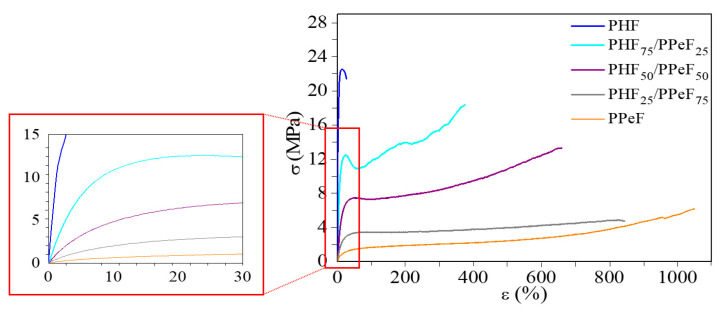
Representative stress–strain curves of PHF, PPeF, and PHF/PPeF blends, with a magnification of the initial elastic region.

The mechanical behaviour of PHF, PPeF, and the blends was analyzed through tensile tests. Figure 4 shows the relative stress–strain curves, while the values of elastic modulus (E), stress (σ_B_), and strain (ε_B_) at break are listed in Table 2.

As commonly accepted, chain flexibility (i.e., T_g_ value) and the degree of crystallinity play a key role in determining mechanical properties [58,59,60]. Considering this, the PHF homopolymer, which is rubbery but highly crystalline, shows the highest elastic modulus (906 MPa) and the lowest elongation at break (below 50%) among the family. Conversely, the amorphous and rubbery PPeF is the material with the lowest elastic modulus (E = 9 MPa) and highest elongation at break (more than 1000%). This is interesting to point out as this homopolymer shows a peculiar behaviour, as it can be processed into a film with elastomeric properties (elongation at break values greater than 1000%) and exhibits an almost instantaneous return to its initial length after break, although amorphous and rubbery at room temperature. As reported in the literature, this evidence can be due to the presence of an ordered phase different from the conventional 3D one, which is responsible for excellent functional properties, including mechanical and barrier ones [52,61,62,63,64]. 

As for the blends, the first main evidence is that the mixing of PHF with PPeF is effective in reducing the stiffness of PHF, as the introduction of increasing amounts of PPeF leads to a progressive decrease in the elastic modulus and stress at break, together with an increase in the elongation at break. More in detail, compared to the values measured for PHF, the values of the elastic modulus decrease approximately 4.4, 10.5, and 28 times in PHF_75_/PPeF_25_, PHF_50_/PPeF_50_, and PHF_25_/PPeF_75_, respectively, while the elongation at break improves by factors of approximately 9, 15, and 19, respectively. This result is remarkable, considering that the two parent homopolymers are mixed only physically together, without any chemical bond among them. Moreover, previous studies carried out on furan-based blends showed poorer elongations at break, not so much higher than those of the most rigid component of the binary blends [43,48]. Therefore, such a behaviour further confirms the good compatibility and the cohesion between two phases, already highlighted by the morphological results (Figure 2). Lastly, the blends PHF_75_/PPeF_25_ and PHF_50_/PPeF_50_ show yielding at strains lower than 20%.

Overall, these results confirm that all the mechanical parameters, such as ductility and flexibility, can be nicely tailored simply by acting on the blend composition, depending on the target properties. Of note, the mechanical properties obtained are absolutely in line with those of commercial plastic materials commonly used for packaging purposes, such as LDPE, LLDPE, and HDPE [65,66].

### 3.5. Evaluation of Gas Barrier Properties 

**Figure 5:**
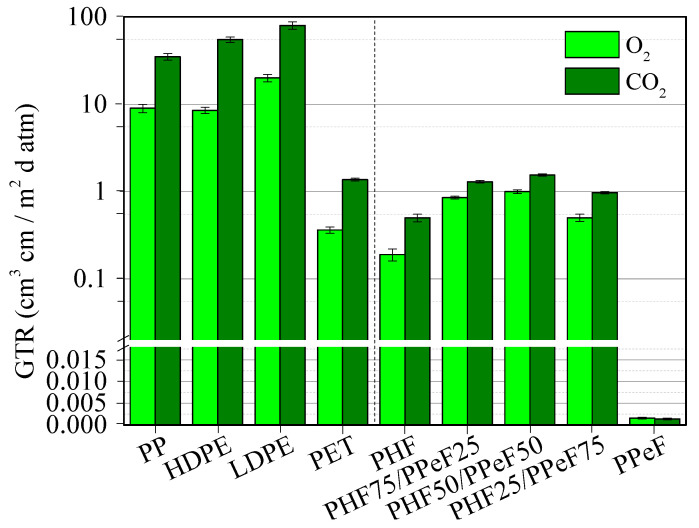
GTR values of PHF, PPeF, and PHF/PPeF blends, together with those of some commonly used packaging materials [72,73].

The analysis of barrier properties is extremely important in understanding the potential applications of the materials under study in the field of food packaging. The permeability tests to pure gasses O_2_ and CO_2_ were carried out at 23 °C and the permeability values, expressed as Gas Transmission Rate (GTR), are reported in Table 2 and shown in Figure 5.

As commonly accepted, keeping the molecular weight of the materials fixed, gas barrier properties are influenced by the free volume fraction, directly related to T_g_, and by the degree of crystallinity. Indeed, glassy polymers have a lower free volume compared to rubbery ones, while 3D crystals hinder the passage of gasses, unlike what occurs in amorphous materials, where the gas molecules can pass more easily [67,68]. However, previous studies have shown that in polymers containing mesogenic units, i.e., materials characterized by the presence of a rigid unit (such as the furan ring) together with a flexible one (such as aliphatic glycols), the formation of a further phase, known as the mesophase, is possible. This peculiar phase is different from the crystalline one and more effective than the latter in reducing the permeability of the materials [61,69]. The formation of the mesophase is favoured in amorphous polymers with T_g_ around room temperature [63,70,71]. Moreover, the presence of crystals within the amorphous phase gives rise to the so-called disclinations, which are channels at the interface between the two phases, through which gasses can pass more easily.

According to this, PHF is the least performing material, despite its T_g_ around room temperature, due to its high crystallinity degree (Table 1). Conversely, PPeF is the material with the lowest GTR values, in which the formation of the mesophase is maximized, thanks to its amorphous and rubbery nature at room temperature. The macromolecular chains at this temperature are indeed unlocked and can rotate and maximize the interchain hydrogen bonds responsible for the formation of the mesophase. According to literature data about similar polymeric systems [48], regarding the PHF/PPeF blends, it can be seen how the GTR values are comparable to those of the PHF homopolymer, as expected on the basis of a conspicuous crystalline portion present in the blends, which develops at the expense of the performing mesophase and which gives rise to disclinations. 

In Figure 5, the GTR values of some commonly used packaging materials (PP, HDPE, LDPE, and PET) [72,73] are also shown for the sake of comparison. Of note, the GTR values of all the materials investigated, including the blends, are comparable (in the case of PET) or even lower (in case of polyolefins) than those of plastic packaging materials, which can be found on the market, making all the materials under study very interesting for applications in the field of food packaging.

### 3.6. Lab-Scale Composting Studies

The capacity of a material to undergo degradation in compost is particularly important in the field of food packaging, with the generated plastic wastes being characterized by a short life-cycle and being difficult to recycle due to contamination with organic matter.

**Figure 6:**
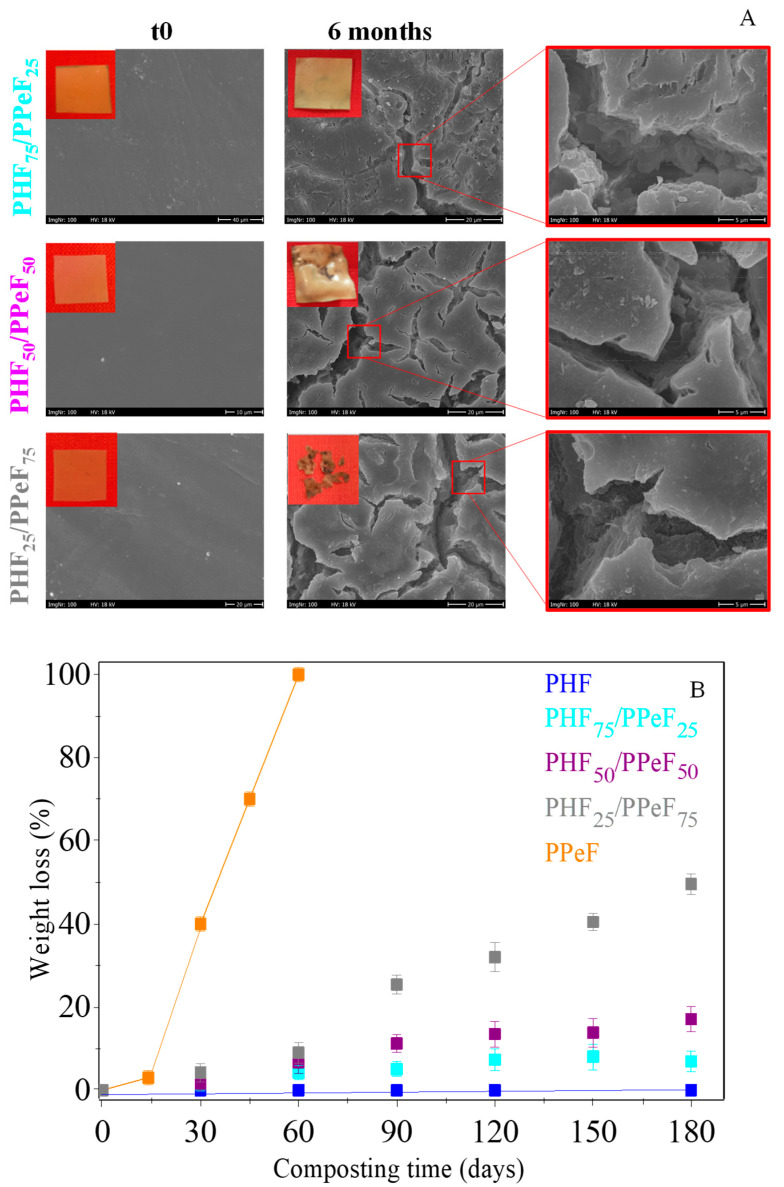
(**A**) Pictures and SEM images of neat (t0) and partially degraded PHF/PPeF blends after 6 months of incubation in compost. (**B**) Gravimetric weight loss as a function of incubation time of PHF, PPeF, and PHF/PPeF blends.

Previous studies from the authors [74,75,76] have indicated that the PHF homopolymer is not degradable in compost, showing negligible weight loss after 6 months of incubation. Conversely, PPeF is compostable, as it completely degrades in about two months. As it can be seen in Figure 6A, the non-incubated films of the mixtures are characterized by a smooth, homogeneous, and semi-transparent surface. On the contrary, the samples incubated for 6 months show opacification, accompanied by an increase in fragility as the amount of PPeF in the blends is higher, suggesting the presence of a higher amount of crystallinity. 

Figure 6B shows the gravimetric weight loss at different incubation times for the mixtures and the relating homopolymers. A direct dependence of weight loss on both the incubation time and the amount of degradable PPeF is clearly evident, although during the explored timescale, none of the samples showed a complete degradation. Indeed, the highest weight loss, of about 50%, was observed in the PHF_25_/PPeF_75_ mixture.

The surface morphology of the polymeric blends was analyzed by means of SEM. As an example, in Figure 6A, the images of the films before and at the end of the composting experiment are shown. As it can be seen, the neat films show a smooth and homogeneous surface, while at the end of the experiment, they undergo significant changes. Even if the weight loss is not complete, deep holes and cracks on the surfaces become visible, whose width and number increase with the amount of PPeF, in agreement with weight loss data. These phenomena are due to both the bulk hydrolytic degradation and the enzymatic activity of the microorganisms inside the compost, which occurs mainly at the surface level. In detail, the hydrolytic cleavage results in the presence of cracks [77,78], while the enzymatic mechanism results in the presence of holes and cavities [79,80]. Considering the results obtained (Figure 6A), it seems that in the blends, the hydrolytic mechanism prevails over the enzymatic one.

**Figure 7:**
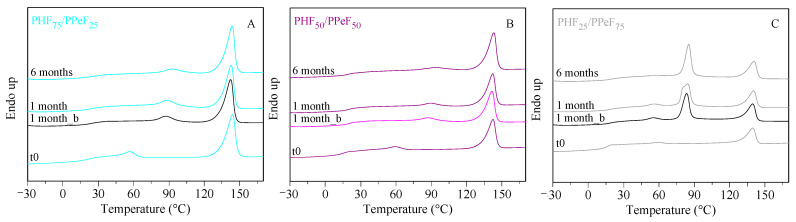
First DSC scans of (**A**) PHF_75_/PPeF_25_, (**B**) PHF_50_/PPeF_50_, and (**C**) PHF_25_/PPeF_75_ after 1 and 6 months of incubation in compost, compared to those of the neat (t0) and blank samples after 1 month of incubation (1 month_b).

In order to verify how the permanence in compost may have influenced the crystallinity and the thermal transitions of the investigated materials, I DSC scans were performed on partially degraded films after 1 and 6 months of incubation. The DSC traces are shown in Figure 7, while the relative thermal data are reported in Table S1.

Taking into account that all the neat samples were semicrystalline, a slight increase in the intensity of the melting phenomenon over time can be observed only in PHF_50_/PPeF_50_. Conversely, the melting temperature remains unchanged, indicating that the main crystalline phase, rich in PHF, was not appreciably attacked.

Of note, an endothermic peak between 85 and 93 °C appeared in all the samples, due to the formation of a less perfect ordered phase compared to the higher melting one, which develops upon the permanence of the samples at 58 °C. In order to understand the origin of this endothermic phenomenon, DSC analysis was also carried out on blank samples. According to Figure 7, the permanence at the incubation temperature seems to be enough to trigger the above-mentioned phenomenon, as the DSC curves of the blanks and composted samples are practically identical (Table S1).

## 4. Conclusions

The realization of bio-based blends with different weight compositions of poly(pentamethylene furanoate) (PPeF) and poly(hexamethylene furanoate) (PHF) has been proved to be an effective way to obtain new sustainable materials in view of applications in the field of flexible food packaging. Indeed, all the starting monomers are bio-based and the synthesis of the homopolymers has been carried out through bulk polycondensation, a solvent-free strategy. In addition, the preparation of the binary mixtures was simple, fast, and easy to scale up to the industrial level.

According to the morphological data, it can be assessed that the blends show a good compatibility, as the homopolymer phases are intimately dispersed, despite the absence of chemical bonds between the macromolecular chains. Blending did not negatively alter the thermal stability, which is one of the strong points of furan polymers. From the mechanical point of view, remarkable improvements with respect to the rigid and brittle PHF were observed, since the presence of increasing amounts of the rubbery and elastomeric PPeF led to a progressive lowering of the stiffness and an increase in the elongation at break, reaching values in line with those of LDPE, LLDPE, and HDPE. This result is particularly remarkable, considering the absence of chemical bonds between the two homopolymers. In addition, the overall barrier properties remained comparable to those of the PHF, and better than those of conventional plastic packaging materials on the market, making the blends very competitive for this application. Lastly, composting experiments did not show complete weight loss, even though high fragmentation in the sample containing the highest amount of PPeF was observed, together with a weight loss of about 50%. Although not fully compostable, the blends, thanks to the similar chemical structure of the starting homopolymers, can be potentially recycled, also resulting in their end-of-life sustainability.

## Figures and Tables

**Table 1:** Thermal (TGA and DSC) and structural characterization data (WAXS) of PHF, PPeF, and PHF/PPeF blends.

	T_onset_ [°C]	T_max_ [°C]	I Scan				II Scan						X_c_ [%]
			T_g_ [°C]	∆c_p_ [J/g°C]	T_m_ [°C]	∆H_m_ [J/g]	T_g_ [°C]	∆c_p_ [J/g°C]	T_cc_ [°C]	∆H_cc_ [J/g]	T_m_ [°C]	∆H_m_ [J/g]	
PHF	374	394	14	0.146	144	39	16	0.156	-	-	144	39	35
PHF_75_/PPeF_25_	373	396	18	0.176	57–144	5.3–37	14	0.373	66	10	143	34	24
PHF_50_/PPeF_50_	375	398	14	0.247	59–142	3.1–27	14	0.425	85	23	142	23	16
PHF_25_/PPeF_75_	376	396	14	0.234	59–140	1.3–13	14	0.366	101	13	139	13	12
PPeF	377	399	16	0.319	-	-	16	0.277	-	-	-	-	0

**Table 2:** Mechanical characterization data and GTR to O_2_ and CO_2_ of PHF, PPeF, and PHF/PPeF blends.

	E (MPa)	σ_B_ (MPa)	ε_B_ (%)	O_2_-TR (cm^3^ cm m^−2^ d^−1^ atm^−1^)	CO_2_-TR (cm^3^ cm m^−2^ d^−1^ atm^−1^)
PHF	906 ± 34	22 ± 1	42 ± 6	0.19	0.5
PHF_75_/PPeF_25_	205 ± 21	16 ± 2	368 ± 12	0.852	1.29
PHF_50_/PPeF_50_	85 ± 4	14 ± 1	657 ± 39	0.997	1.55
PHF_25_/PPeF_75_	32 ± 3	5 ± 1	803 ± 43	0.505	0.969
PPeF	9 ± 1	6 ± 1	1050 ± 200	0.0016	0.0014

## Data Availability

The raw data supporting the conclusions of this article will be made available by the authors on request.

## Supplementary Materials

The following supporting information can be downloaded at: https://www.mdpi.com/article/10.3390/polym16162342/s1, Figure S1. ^1^H-NMR spectra of PPeF (top) and PHF (bottom) homopolymers, with peaks attribution; Figure S2. ^13^C-NMR spectra of PPeF (top) and PHF (bottom) homopolymers, with peaks attribution; Table S1. I scan DSC data of partially degraded PHF/PPeF blends.
