# Supplementary material for: Fully Bio-Based Blends of Poly (Pentamethylene Furanoate) and Poly (Hexamethylene Furanoate) for Sustainable and Flexible Packaging
Source: Polymers (Basel). 2024 Aug 19;16(16):2342. doi: 10.3390/polym16162342 (PMC11360354; doi:10.3390/polym16162342)
Supplement: Supplementary file 1 [file polymers-16-02342-s001.zip › polymers-3153090-supplementary-update.pdf]

Supplementary Information

Fully biobased blends of poly(pentamethylene furanoate) and poly(hexamethylene furanoate) for sustainable and flexible packaging

Giulia Guidotti[1], Arianna Palumbo[1], Michelina Soccio[1], Massimo Gazzano[2], Elisabetta Salatelli[3], Valentina Siracusa[4], Nadia Lotti[1,5]

[1] *Department of Civil, Chemical, Environmental, and Materials Engineering, University of Bologna, Via Terracini 28, Bologna, Italy;*

[2] *Institute for Organic Synthesis and Photoreactivity, ISOF-CNR, Via Gobetti 101, Bologna, Italy;*

[3] *Departmet of Industrial Chemistry "Toso Montanari", University of Bologna, Viale Risorgimento 4, Bologna, Italy;*

[4] *Chemical Science Department, University of Catania, Viale A. Doria 6, Catania, Italy;*

[5] *Interdepartmental Center for Industrial Agro-Food Research, CIRI-AGRO, Via Q. Bucci 336, Cesena, Italy.*

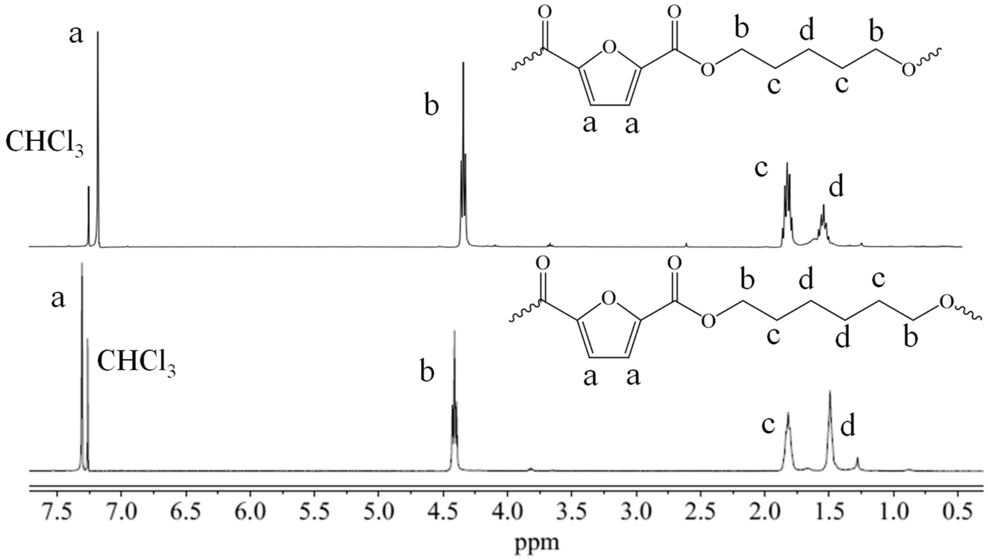

Figure S1. ^{1}H-NMR spectra of PPeF (top) and PHF (bottom) homopolymers, with peaks attribution.

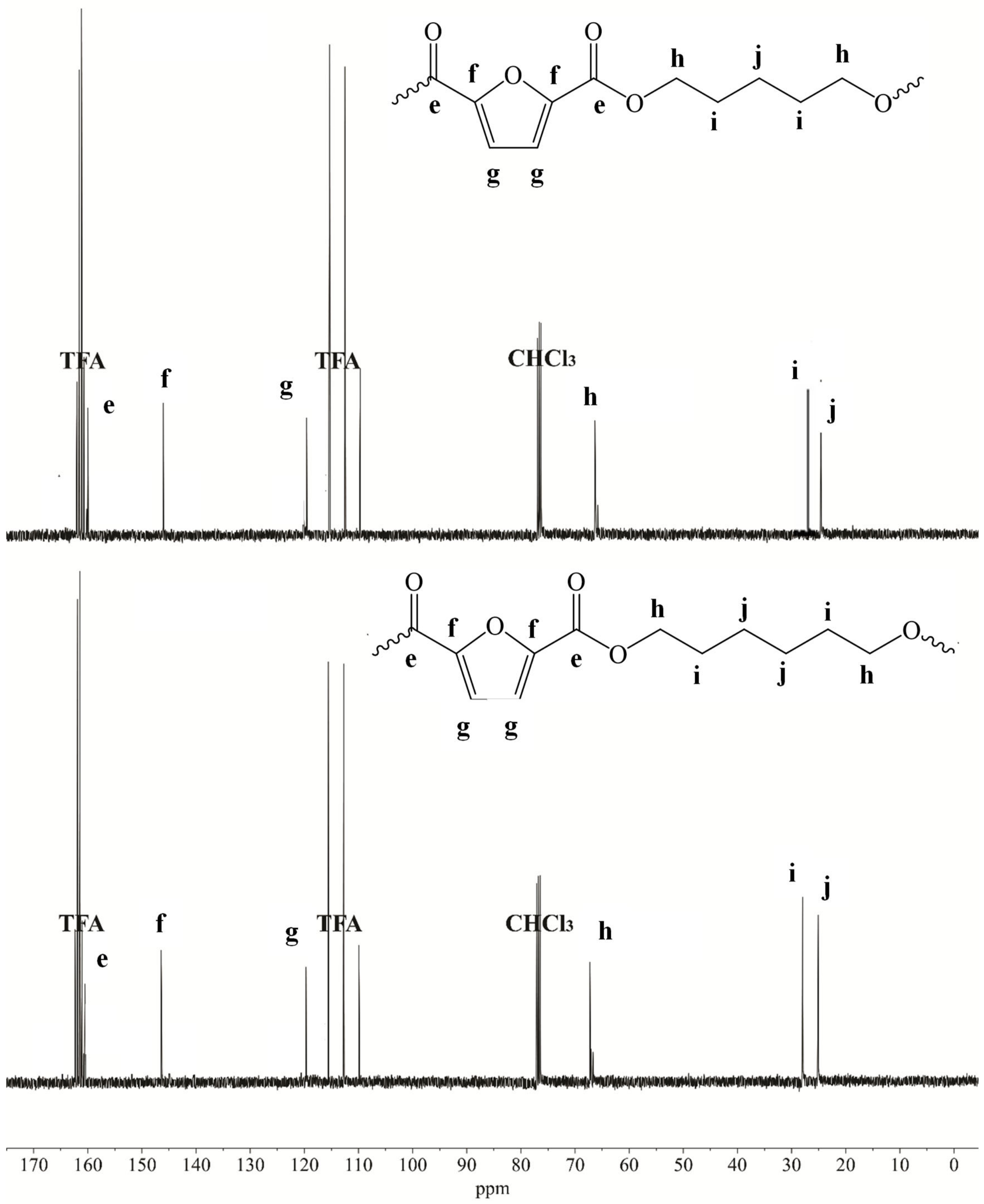

Figure S2. ^{13}C-NMR spectra of PPeF (top) and PHF (bottom) homopolymers, with peaks attribution..

Table S1. I scan DSC data of partially degraded PHF/PPeF blends.

	**$PHF_{75}/PPeF_{25}$**			**$PHF_{50}/PPeF_{50}$**			**$PHF_{25}/PPeF_{75}$**		
	t0	1 month	6 months	t0	1 month	6 months	t0	1 month	6 months
**T_g (°C)**	22	24	26	15	21	20	15	19	19
**ΔC_p (J/g°C)**	0.176	0.204	0.210	0.247	0.131	0.157	0.234	0.116	0.101
**T_m (°C)**	57<br>144	89<br>143	93<br>144	59<br>142	89<br>142	93<br>143	59<br>140	55<br>84<br>140	85<br>140
**ΔH_m (J/g)**	5.3<br>37	7.4<br>34	5.5<br>38	3.3<br>23	3.0<br>26	4.0<br>30	1.3<br>13	1.7<br>17<br>13	18<br>14
